# Tricin Isolated from Enzyme-Treated *Zizania latifolia* Extract Inhibits IgE-Mediated Allergic Reactions in RBL-2H3 Cells by Targeting the Lyn/Syk Pathway

**DOI:** 10.3390/molecules25092084

**Published:** 2020-04-29

**Authors:** Jae-Yeul Lee, Se-Ho Park, Kwang-Hwan Jhee, Seun-Ah Yang

**Affiliations:** 1 Institute of Natural Science, Keimyung University, Daegu 42601, Korea; sunriseow@naver.com (J.-Y.L.); p86ks1@naver.com (S.-H.P.); 2 Department of Applied Chemistry, Kumoh National Institute of Technology, Gumi 39177, Korea; khjhee@kumoh.ac.kr; 3 Department of Food Science and Technology, Keimyung University, Daegu 42601, Korea

**Keywords:** tricin, enzyme-treated *Zizania latifolia* extract, anti-allergic activity, FcεRI signaling pathway, MAPK signaling pathway, arachidonic acid signaling pathway

## Abstract

Tricin, a flavone present in rice bran, is confirmed as the major efficacious compound present in the enzyme-treated *Zizania latifolia* extract (ETZL), which protects against UVB-induced skin-aging. However, the suppressive mechanism of tricin on allergic responses remains unknown. The present study, therefore, aimed to determine the mechanisms of tricin and ETZL on mast cell degranulation in IgE-activated rat basophilic leukemia cell line (RBL-2H3) cells. We investigated the regulatory effects of tricin and ETZL on degranulation, production of cytokines and lipid mediators, and signaling proteins involved in the IgE-bound high-affinity IgE receptor activation, mitogen-activated protein kinase, arachidonic acid and Syk. The production of β–hexosaminidase, tumor necrosis factor-α, interleukin-4, leukotrienes (LT) B_4_, LTC_4_ and prostaglandin E_2_ in IgE-stimulated RBL-2H3 cells were significantly inhibited by exposure to tricin or ETZL. Moreover, tricin and ETZL inhibit the phosphorylation of cytosolic phospholipase A2, 5-lipoxygenase and cyclooxygenase-2. Furthermore, the phosphorylation of Akt, ERK, p38, JNK, protein kinase Cδ and phospholipase Cγ1 were effectively suppressed by both samples. Exposure to tricin or ETZL also significantly decreases the phosphorylation of Lyn and Syk, but has minimal effect on Fyn. Taken together, our data indicate that tricin and ETZL are potential anti-allergic materials that could be applied for the prevention of allergy-related diseases.

## 1. Introduction

*Zizania latifolia* (Gramineae) is the only member of the wild rice genus *Zizania* native to Asia, including China, Korea and Japan. *Z. latifolia* Turcz differs botanically from the other wild rice species, including *Z. aquatica, Z. palustris* and *Z. texana*. The *Z. latifolia* grains impart numerous pharmacological effects, including suppression of hyperlipidemia [1], reduction of oxidative stress in cells [2], decreasing blood glucose levels [3], improvement of insulin resistance [3] and anti-obesity [1]. Recently, it has been reported that the aerial portion of *Z. latifolia* exhibits a variety of inhibitory activities, such as H_2_O_2_-induced apoptosis in Neuro2A cells [4], angiotensin-converting enzyme and oxidative stress [5] and ultraviolet (UV) B-induced wrinkle formation in SKH-1 hairless mice and in human dermal fibroblasts [6,7]. The methanol extract of the aerial portion suppresses allergic responses in rat basophilic leukemia cell line (RBL-2H3) through inhibition of compound 48/80-induced degranulation, antigen-induced β-hexosaminidase release and phorbol 12-myristate 13-acetate plus A23187-induced tumor necrosis factor (TNF)-α production [8].

**Figure 1:**
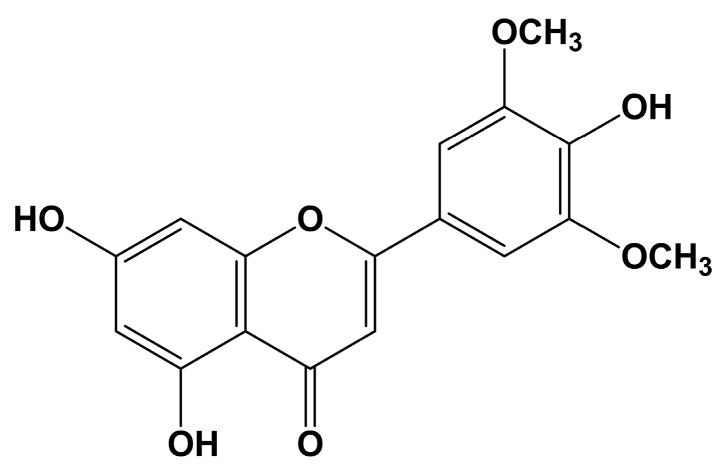
Chemical structure of tricin.

In our previous study, we identified five derivatives of tricin (4′,5,7-trihydroxy-3′,5′-dimethoxyflavone) (Figure 1) in the methanol extract prepared using the aerial portion of *Z. latifolia* [8]. The tricin derivatives were determined to prevent release of β-hexosaminidase in RBL-2H3 cells sensitized with anti-dinitrophenyl (DNP) immunoglobulin E (IgE). Exposure to tricin is known to suppress cyclooxygenase (COX) activity in mice, and prostaglandin E_2_ (PGE_2_) production in colon cells and murine plasma [9]. Tricin was found to be the quantitatively major component obtained in 70% ethanol extract of *Z. latifolia*. Treatment with an enzyme mixture significantly increased the tricin content in a hydrolyzed ethanol extract [7].

Mast cells play a major role in IgE-mediated allergic responses (hypersensitivity) by producing inflammatory mediators such as histamine, leukotrienes, prostaglandins and cytokines upon binding of an antigen to the IgE-bound high-affinity IgE receptor (FcεRI) [10]. Since histamine released from cytosolic granules in mast cells induces acute allergic responses, antihistamines are commonly used to treat allergic diseases. Arachidonic acid metabolites (such as leukotrienes) and cytokines (including TNF-α and interleukin (IL)-4) are also critical in prompting allergic reactions [10]. Leukotrienes (LT) such as LTB_4_ and LTC_4_ attract neutrophils and stimulate the late-phase allergic reaction [11]. Cytokines released by a T-helper type 2 (Th_2_) response (such as IL-4, IL-6 and IL-13) activate B cells to produce IgE, thereby activating mast cells and inducing allergic inflammation [12].

In addition, FcεRI stimulation results in the immediate activation of two Src family kinases, Lyn and Fyn, and a tyrosine kinase spleen tyrosine kinase (Syk), to initiate mast cell degranulation, with subsequent activation of the phospholipase Cγ (PLCγ) and protein kinase B (Akt) signaling pathways [10,13]. The underlying pathophysiologic mechanisms of allergic response are well-documented by previous in vitro and in vivo studies [14]. A mitogen-activated protein kinase (MAPK) cascade is an important signaling pathway that regulates the differentiation, activation, proliferation, degranulation and migration of immune cells, including mast cells. The p38 MAP kinase activates IL-4 in bone marrow mast cells, and the activation of c-Jun N terminal kinase (JNK) is also responsible for the production of pro-inflammatory cytokines in mast cells, including IL-2, IL-6 and TNF-α [15]. The phosphorylated Akt regulates the transcription of IL-2 and TNF-α promoters [12]. Moreover, the overexpression of Syk activates extracellular signal-regulated kinase 1/2 (ERK) phosphorylation, and hence the arachidonic acid signaling pathway, including activation of the expressions of cytosolic phospholipase A2 (cPLA_2_), 5-lipoxygenase (5-LO) and COX-2, and secretions of leukotrienes and prostaglandins [11,12]. Phosphorylation of ERK1/2 plays a role in signaling TNF-α, IL-3, IL-5 and IL-13 secretion in mast cells [12,15]. Therefore, decreasing secretions of cytokines (TNF-α, IL-4), leukotrienes and prostaglandins, is crucial in anti-allergic reactions.

Antihistamines and mast cell stabilizers are widely used as anti-allergy agents for ameliorating allergic inflammatory symptoms, despite some side-effects such as drowsiness. Recently, efficacious edible plant materials devoid of any side effects are gaining attention as safe anti-allergic agents. RBL-2H3 cells express FcεRI on the cell membrane, and the IgE-bound FcεRI can be activated by antigens; thus, these cells are widely used for in vitro investigations on the mechanism of mast cell activation [16,17].

Mechanisms associated with the anti-allergic action of tricin isolated from enzyme-treated *Z. latifolia* extract (ETZL) remain unclear. Therefore, to understand the mechanism by which tricin or ETZL alleviates the allergic response in IgE-mediated allergic reactions in RBL-2H3 cells, this study investigated the regulatory effects on signaling cascades, such as FcεRI, arachidonic acid and MAPKs.

## 2. Results and Discussion

### 2.1. Effects of the Non-Enzyme Treated Ethanol Extract of Z. latifolia (NEZL), ETZL and Tricin on Anti-DNP IgE-Mediated Degranulation in RBL-2H3 Cells

As reported in our previous studies, the methanol extract from aerial *Z. latifolia* exerts excellent anti-degranulation activity in RBL-2H3 cells, and contains tricin and its derivatives as the major components for anti-allergic activity. The identified derivatives include tricin-7-*O*-β-d-glucopyranose, tricin-4’-*O*-(*threo*-β-guaiacylglyceryl) ether 7-*O*-β-d-glucopyranose, tricin-4’-*O*-(*erythro*-β-guaiacylglyceryl) ether 7-*O*-β-d-glucopyranose, tricin-4’-*O*-(*threo*-β-guaiacylglyceryl) ether 7’’-*O*-β-d-glucopyranose and tricin-4’-*O*-(*erythro*-β-guaiacylglyceryl) ether 7’’-*O*-β-d-glucopyranose [8,18]. The same derivatives were also identified for the ethanol extract of *Z. latifolia* (data not published). Additionally, an enzymatic treatment applied to augment the amount of tricin increased the content by 1.27-fold in ETZL as compared to non-enzyme treated ethanol extract (NEZL), giving 25.0 mg/100 g dried *Z. latifolia* with extraction yield of 17.45%, estimating 0.14% tricin in ETZL [6]. Our previous quantitative study identifying the tricin derivatives in ETZL confirmed the simultaneous increase of four derivatives along with tricin levels, as compared to the contents obtained in NEZL (manuscript in preparation). Based on these data, we concluded that the commercially available major compound tricin, and not its derivatives, is the active component and is also the most appropriate and potent compound to be applied as a marker for industrial quality control of the extract of *Z. latifolia* (ETZL). 

**Figure 2:**
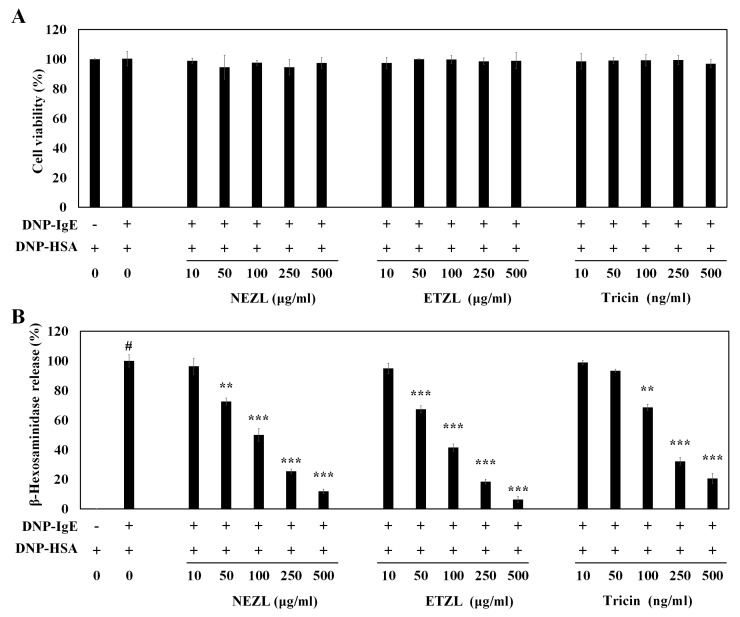
Effects of tricin and enzyme-treated *Zizania latifolia* extract (ETZL) on cell viability and degranulation in IgE-stimulated rat basophilic leukemia (RBL-2H3) cells. (**A**) Cell viability; (**B**) β-hexosaminidase. RBL-2H3 cells were seeded in a 24-well plate (1 × 10^5^ cells/well) overnight at 37 °C, and further incubated with anti-dinitrophenyl (DNP)-IgE (0.05 μg/mL) for 24 h. The IgE-sensitized cells were preincubated with varying concentrations of tricin (10–500 ng/mL) or ETZL (10–500 μg/mL) for 1 h, and subsequently stimulated with dinitrophenyl-human serum albumin (DNP-HSA) (0.1 μg/mL) for 4 h. All values are the mean ± S.E.M. of three independent experiments. Values of ** *p* < 0.01 and *** *p* < 0.001 were considered significantly different to the anti-DNP IgE plus DNP-HSA, unpaired Student’s *t*-test. ^#^ *p* < 0.001 compared to the control.

Thus, to investigate the mechanistic action of tricin in an anti-allergic reaction, we first evaluated the effects of NEZL, ETZL and tricin isolated from ETZL on allergic responses in the rat mast cell line RBL-2H3. In the present study, 0.1% tricin was used for each concentration of ETZL to check the efficacy of ETZL derived from tricin. Anti-DNP IgE-sensitized RBL-2H3 cells were exposed to varying concentrations of tricin and ETZL, and degranulation was triggered by adding dinitrophenyl-human serum albumin (DNP-HSA) as antigen. The effects of NEZL, ETZL and tricin were determined on the cell viability by the MTT assay. As shown in Figure 2A, exposure of an equal number of viable cells to NEZL, ETZL and tricin exhibits no cytotoxicity at any of the tested concentrations (*p* > 0.05). Effects on the IgE-mediated degranulation were then evaluated by determining the activity of β-hexosaminidase, a biomarker of histamine release, in IgE-activated RBL-2H3 cells. As expected, NEZL exhibit lower inhibitory activity against the release of β-hexosaminidase (50% inhibitory concentration (IC_50_), 106.79 μg/mL), as compared to ETZL (IC_50_, 70.79 μg/mL) (*p* < 0.001) (Figure 2B). In addition, tricin inhibited the release of β-hexosaminidase in a concentration-dependent manner with an IC_50_ value of 184.84 ng/mL (Figure 2B). These data indicate that the inhibitory effects are not the result of the cytotoxicity, and the degranulation-inhibitory activity of ETZL is mainly exerted by the major component, tricin. 

Monitoring of mast cell degranulation using the β-hexosaminidase assay in RBL-2H3 cells is widely used to determine the effectiveness on anti-allergic responses, and is a convenient in vitro cell model for studying new molecules that inhibit mast cell activation or degranulation [19,20]. Histamine, secreted with β-hexosaminidase from mast cells, induces allergic inflammation, resulting in chronic allergic symptoms such as atopy, asthma, etc. Recent studies have reported the suppressive effects of flavonoids [21,22] and plant extracts [17] on mast cell degranulation. Among the various bioactive plant materials, *Actinidia arguta* extract has been recognized as a functional ingredient in facilitating the prevention of atopy, food allergy and asthma [23,24,25].

### 2.2. Effects of Tricin and ETZL on the Secretion of Cytokines and Lipid Mediators

**Figure 3:**
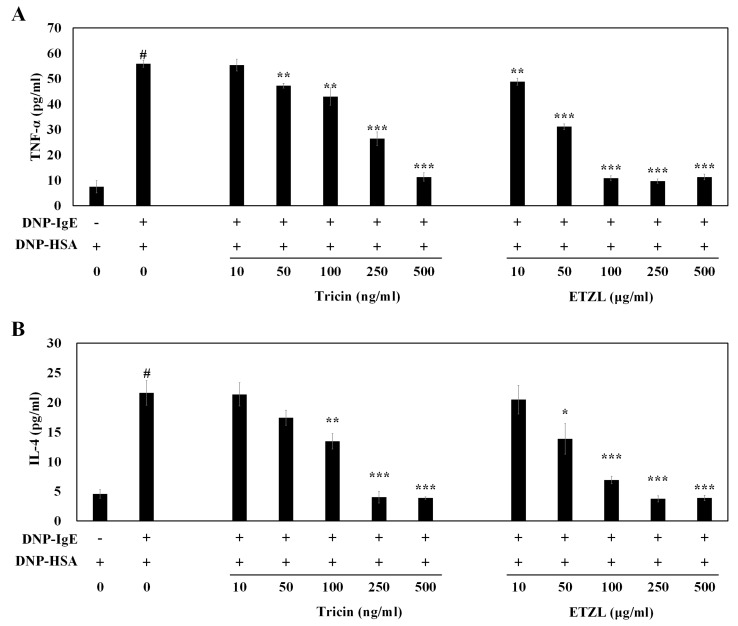
Effect of tricin and ETZL on pro-inflammatory cytokines in IgE-mediated RBL-2H3 cells. (**A**) Tumor necrosis factor (TNF)-α secretion; (**B**) IL-4 secretion. IgE-sensitized RBL-2H3 cells were pre-incubated with tricin or ETZL for 1 h before antigen treatment for 4 h. All values are the mean ± S.E.M. of three independent experiments. Values of * *p* < 0.05, ** *p* < 0.01 and *** *p* < 0.001 were considered significantly different to the anti-DNP IgE plus DNP-HSA, unpaired Student’s *t*-test. ^#^ *p* < 0.001 compared to the control.

Following the immediate histamine release subsequent to antigen-mediated FcεRI aggregation, degranulation of mast cells results in the secretion of inflammatory mediators, including TNF-α, IL-4, leukotrienes and prostaglandins, which play key roles during the late phase of inflammatory reactions [16,17]. Inhibiting these mediators from activated mast cells is considered a promising strategy to ameliorate allergic responses. Thus, we examined the effects of tricin and ETZL on the release of inflammatory cytokines (TNF-α and IL-4) by ELISA in anti-DNP-IgE-stimulated RBL-2H3 cells. As presented in Figure 2, the ETZL, containing a 1.27-fold higher tricin content, exhibited a 1.51-fold higher inhibitory activity than NEZL. We therefore decided to perform the subsequent experiments using tricin and ETZL, and not NEZL. Both TNF-α and IL-4 secretions were increased by antigen stimulation, to 55.8 pg/mL and 21.6 pg/mL (*p* < 0.001), respectively. The TNF-α production was inhibited after exposure to tricin (55.3, 47.2 and 43.0 pg/mL at 10, 50 and 100 ng/mL, respectively) and ETZL (48.8, 31.1 and 10.8 pg/mL at 10, 50 and 100 μg/mL, respectively) in dose-dependent manners (Figure 3A). Similarly, IL-4 secretion was also suppressed by tricin (21.4, 17.4, 13.4 pg/mL at 10, 50 and 100 ng/mL, respectively) and ETZL (20.5, 13.9 and 6.9 pg/mL at 10, 50 and 100 μg/mL, respectively) (Figure 3B). Similar to the result of Figure 2, the release-suppressive effect of ETZL (*p* < 0.001) was higher than tricin (*p* < 0.01) at 100 µg/mL, indicating that apart from tricin, ETZL may have other bioactive compounds imparting the anti-allergic inflammation, including derivatives of tricin and other trace amounts, as shown in our previous reports [8].

The histamine released from mast cells increases the expression level of FcεRI on mast cells, thereby stimulating the cells for further antigen-induced activation, resulting in the production of inflammatory cytokines and lipid mediators. Recent studies have determined that apart from early-phase allergic responses, mast cells also contribute to the late-phase reactions by modulation of cytokines and mediators [17]. TNF-α, a potent inflammatory cytokine, is produced in activated macrophages and T cells, as well as antigen-stimulated mast cells [11]. IL-4 secreted from mast cells induces the production of IgE in B cells and Th_2_ cell differentiation, which subsequently causes anaphylaxis [11]. Several active flavonoids, such as kaempferol [26], quercetin [26], luteolin [26], chalcone [27] and curcumin [28], inhibit the antigen-induced degranulation, and the production of TNF-α and IL-4 in IgE-sensitized RBL-2H3 cells; these molecules have therefore been suggested as effective applications for the prevention of immediate-phase as well as late-phase reactions.

**Figure 4:**
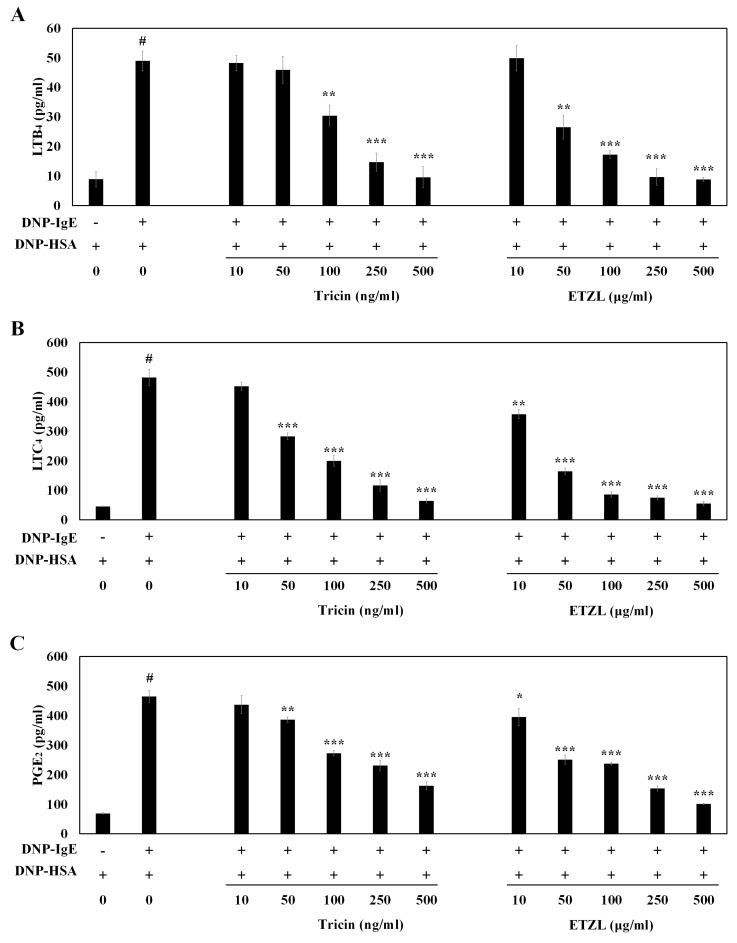
Inhibitory effect of tricin and ETZL on formation of leukotrienes (LT)B_4_, LTC_4_ and prostaglandin E_2_ (PGE_2_) in RBL-2H3 cells stimulated by an IgE–antigen complex. (**A**) LTB_4_ secretion; (**B**) LTC_4_ secretion; (**C**) PGE_2_ secretion. RBL-2H3 cells were seeded in a 24-well plate and cultured overnight at 37 °C; cells were subsequently washed and further incubated with anti-DNP-IgE (0.05 μg/mL) for 24 h. The cells were incubated with tricin (10–500 ng/mL) or ETZL (10–500 μg/mL) for 1 h, followed by stimulation with DNP-HSA (0.1 μg/mL) for 4 h. All values are the mean ± S.E.M. of three independent experiments. Values of * *p* < 0.05, ** *p* < 0.01 and *** *p* < 0.001 were considered significantly different to the anti-DNP IgE plus DNP-HSA, unpaired Student’s *t*-test. ^#^ *p* < 0.001 compared to the control.

Conversely, the activation of arachidonate cascade in IgE-stimulated mast cells induces the production of pro-inflammatory lipid mediators such as LTB_4_, LTC_4_ and PGE_2_, which initiate chronic inflammation in allergy-related diseases [17]. We therefore evaluated the effects of tricin and ETZL on the production of LTB_4_, LTC_4_ and PGE_2_. Preincubation with varying concentrations of tricin or ETZL prior to antigen stimulation markedly suppresses the production of LTB_4_ (30.4 pg/mL at 100 ng/mL tricin, 17.2 pg/mL at 100 μg/mL of ETZL), LTC_4_ (200.0 pg/mL at 100 ng/mL of tricin, 86.3 pg/mL at 100 μg/mL of ETZL) and PGE_2_ (273.0 pg/mL at 100 ng/mL of tricin, 237.2 pg/mL at 100 μg/mL of ETZL) (*p* < 0.001) (Figure 4A–C). These results indicate that tricin as well as the tricin-containing ETZL inhibit leukotriene as well as prostaglandins synthesis, thereby preventing allergic inflammation induced by LTB_4_, LTC_4_ or PGE_2_.

**Figure 5:**
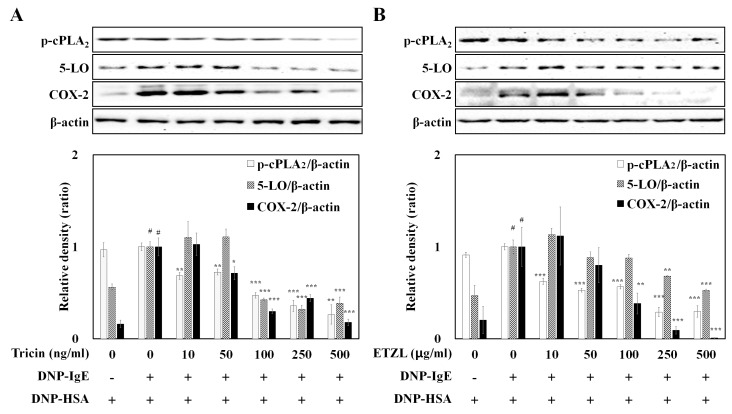
Effect of (**A**) tricin or (**B**) ETZL on phosphorylation of rate limiting enzymes in the arachidonate cascade. The expressions of p-cytosolic phospholipase A2 (cPLA_2_), 5-lipoxygenase (5-LO), COX-2 and β-actin were determined as described in the Western blot method. All values are the mean ± S.E.M. of three independent experiments. Values of * *p* < 0.05, ** *p* < 0.01 and *** *p* < 0.001 were considered significantly different to the anti-DNP IgE plus DNP-HSA, unpaired Student’s *t*-test. ^#^ *p* < 0.001 compared to the control.

We further investigated the effects of tricin and ETZL on the activation of enzymes involved in the synthesis of eicosanoids, including cPLA_2_, 5-LO and COX-2, in IgE-activated RBL-2H3 cells. Both samples inhibit the phosphorylation of cPLA_2_, 5-LO and COX-2, as seen in Figure 5A,B, suggesting that tricin and ETZL suppress the formation of arachidonate metabolites through inhibition of 5-LO (an early enzyme in leukotriene synthesis) and COX-2 expression (the rate-limiting enzyme in prostaglandin synthesis). 

Phosphorylation of cPLA_2_ generates arachidonic acid (a potent metabolic precursor) from phospholipid; subsequently, the arachidonic acid metabolizing enzymes (COX-2 and 5-LO) produce PGE_2_ and LTs, respectively, from arachidonic acid. Numerous studies have reported that various compounds (including flavonoids, alkaloids, terpenoids and saponins) are natural inhibitors of arachidonic acid metabolite formation and the metabolite-mediated allergic inflammation [29]. Tricin from *Oryza sativa* L. is also reported as an anti-inflammatory molecule for COX-2-mediated PGE_2_ production in LPS-stimulated human peripheral blood mononuclear cells [30] as well as in human colon-derived human colon epithelial cell or HCA-7 cells (in vitro) and in *Apc^Min^* mice (in vivo) [9]. Our data also indicate that tricin isolated from ETZL is a natural inhibitor of allergic inflammatory LTs and PGs.

### 2.3. Effects of Tricin and ETZL on the MAPK Signaling Pathway

**Figure 6:**
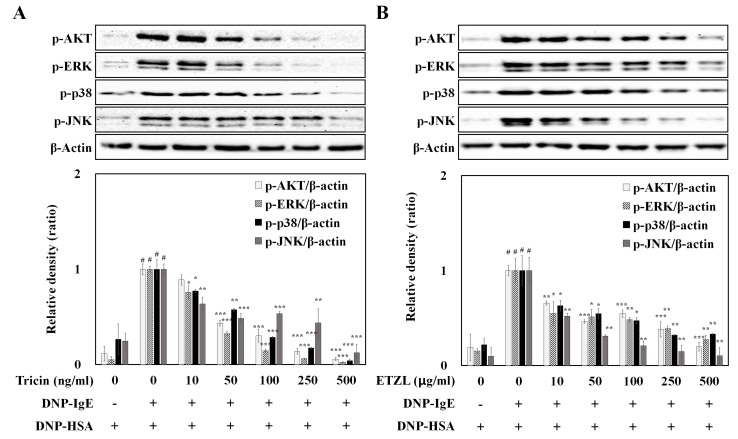
Effect of (**A**) tricin and (**B**) ETZL on phosphorylation of proteins in the mitogen-activated protein kinase (MAPK) signaling cascade, in IgE-activated RBL-2H3 cells. IgE-sensitized RBL-2H3 cells were exposed to tricin or ETZL for 1 h, and subsequently stimulated by antigen for 10 min. All values are the mean ± S.E.M. of three independent experiments. Values of * *p* < 0.05, ** *p* < 0.01 and *** *p* < 0.001 were considered significantly different to the anti-DNP IgE plus DNP-HSA, unpaired Student’s *t*-test. ^#^ *p* < 0.001 compared to the control.

Since activation of MAPKs plays major roles in the formation of inflammatory cytokines, we next examined the effects of tricin and ETZL on the Akt and MAPK signaling pathways in IgE-stimulated RBL-2H3 cells [10]. The phosphorylation of Akt, p38 and JNK induces the expression of proinflammatory cytokines such as TNF-α and IL-4, and the phosphorylation of ERK activates cPLA_2_ to synthesize arachidonic acid metabolites by 5-LO or COX-2, inducing the secretions of LTB_4_, LTC_4_ or PGE_2_. Akt is a downstream effector of Syk, and the phosphorylation of Akt is involved in mast cell degranulation [14]. Proteins involved in the MAPK signaling cascade were phosphorylated upon IgE-mediated mast cell activation. However, exposure to tricin and ETZL inhibited the phosphorylation of Akt and MAPKs (such as ERK, p38 and JNK) that regulate the cytokine expression (Figure 6A,B). This indicates that tricin and ETZL directly block the activation of Syk-dependent signaling, thereby preventing the secretions of cytokines (TNF-α and IL-4) as well as arachidonate metabolites (LTB_4_, LTC_4_ and PGE_2_). 

### 2.4. Effects of Tricin and ETZL on the Activation of the FcεRI Signaling Pathway

**Figure 7:**
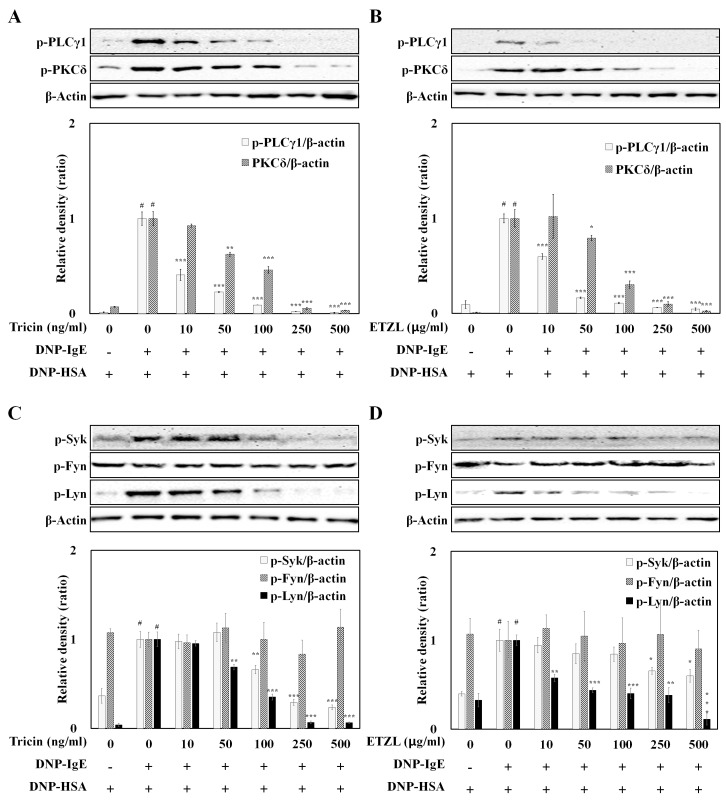
Inhibitory effect of tricin and ETZL on phosphorylation of proteins in the FcεRI signaling cascade in RBL-2H3 cells stimulated by an IgE–antigen complex. RBL-2H3 cells were seeded overnight in a 24-well plate at 37 °C; cultured cells were washed and further incubated with anti-DNP-IgE for 24 h. The cells were then incubated with varying concentrations of (**A**,**C**) tricin (10–500 ng/mL) or (**B**,**D**) ETZL (10–500 μg/mL) for 1 h, and subsequently stimulated by DNP-HSA for 4 h. All values are the mean ± S.E.M. of three independent experiments. Values of * *p* < 0.05, ** *p* < 0.01 and *** *p* < 0.001 were considered significantly different to the anti-DNP IgE plus DNP-HSA, unpaired Student’s *t*-test. ^#^ *p* < 0.001 compared to the control.

Upon binding of an antigen to the IgE-bound FcεRI, the signaling pathways involving PI3K/PLCγ/protein kinase C (PKC) and MAPKs are activated. The phosphorylation of PLCγ1 and PKCδ stimulates the secretion of histamine along with β-hexosaminidase and inflammatory cytokines, resulting in allergic reactions [8,31]. The phosphorylation of Lyn, Fyn and Syk is activated immediately upon FcεRI stimulation, and are known to play essential roles in the initiation of mast cell degranulation and activation [10,13,17]. Thus, down-regulating the phosphorylation of Syk and PLCγ blocks the FcεRI-mediated mast cell activation [16]. PLCγ also plays a key role in the generation of inositol-1,4,5-triphosphate to induce intracellular Ca^2+^ mobilization, which is a crucial step for mast cell degranulation [32,33]. Thus, we performed by Western blot to evaluate the effects of tricin and ETZL on activation of the IgE-FcεRI pathway in the mast cell line. Both tricin and ETZL significantly suppressed the phosphorylation of PLCγ1 and PKCδ (Figure 7A,B), thereby inhibiting the release of β-hexosaminidase and inflammatory cytokines in activated RBL-2H3 cells (Figure 2B).

To investigate the regulatory effects of tricin and ETZL on the early stage of FcεRI signaling pathway, we next examined the phosphorylation levels of Fyn, Lyn and Syk in IgE-activated RBL-2H3 cells. The phosphorylation levels of Lyn and Syk were up-regulated by IgE-stimulation (2.37- and 2.56-fold, respectively, vs. untreated control), but Fyn levels were minimally altered (Figure 7C,D). Tricin and ETZL also down-regulated the enhanced expressions of Lyn (0.35- and 0.40-fold of IgE-treated control, respectively) and Syk (0.57- and 0.84-fold of IgE-treated control, respectively), but not Fyn (1.00- and 0.97-fold of IgE-treated control), suggesting that tricin and tricin-containing ETZL effectively prevent the IgE-mediated allergic reactions through inhibition of Syk-Lyn, which subsequently suppresses the activation of PLCγ and MAPK signaling in RBL-2H3 cells. 

Among the numerous natural resources known to inhibit Syk and MAPK activation in mast cells, saponins obtained from roots of *Platycodon grandiflorum* and *Loranthus parasiticus* extract have been reported to inhibit allergic reactions by suppressing the phosphorylation of Akt, ERK, p38 and JNK, as well as inhibiting Syk and Lyn in antigen-stimulated RBL-2H3 cells, suggesting possible therapeutic applications in allergy related diseases [17,34].

In conclusion, the present study demonstrates that ETZL exerts anti-allergic effects in IgE-stimulated mast cells, and tricin is responsible for its preventive action against the allergic reactions. The suppressive mechanism of tricin involved in the signaling cascade necessary for IgE-mediated allergic reactions, were investigated via the FcεRI signaling pathway related to mast cell degranulation and secretions of cytokines and chemo-attractants. Tricin inhibits the release of β-hexosaminidase, production of proinflammatory cytokines TNF-α and IL-4 through the inhibition of PLCγ/PKCδ and MAPKs pathways, and also the synthesis of arachidonate metabolites LTB_4_, LTC_4_ and PGE_2_ by affecting the enzyme activities of 5-LO and COX-2 as well as ERK and cPLA. Furthermore, our data demonstrates that phosphorylation of Lyn and Syk proteins involved in the early stage of FcεRI signaling pathway are effectively suppressed by tricin. In the current study, the numerous reactions that inhibit allergic responses after exposure to tricin were investigated at various phosphorylation steps in the signaling pathways triggered by antigen bound IgE-FcεRI on mast cells. Further studies are required to determine the in vivo efficacy of tricin containing ETZL, using allergy animal models for asthma, allergic rhinitis and atopic dermatitis. Overall, our data confirms that tricin and ETZL possess potential anti-allergic activity, and tricin can be used as a phytochemical to inhibit the mast cell-derived mediator release, in the development of functional products related to allergic disorders.

## 3. Materials and Methods 

### 3.1. Reagents

3-(4,5-dimethylthiazol-2-yl)2-,5-diphenyltetrazolium bromide (MTT), dinitrophenyl-human serum albumin (DNP-HSA), anti-DNP-IgE and 4-nitrophenyl *N*-acetyl-β-d-glucosaminide (p-NAG) were purchased from Sigma Aldrich Co. (St. Louis, MO, USA). Antibodies against phospho-protein kinase B (Akt; #9271), phospho-cPLA_2_ (#2831), phospho-extracellular signal-regulated kinase 1/2 (ERK; #9101), phospho-c-Jun N terminal kinase 1/2 (JNK; #9251), phospho-Src family protein kinase (Lyn; #2731), phospho-p38 (#9211), phospho-PKC*δ* (#2055), phospho-PLC*γ*1 (#2821), phospho-spleen tyrosine kinase (Syk; #2710), horseradish peroxidase-linked anti-rabbit secondary antibody (#7074) and anti-mouse secondary antibody (#7076) were purchased from Cell Signaling Technology (Beverly, MA, USA). Specific antibodies against phospho-Fyn (orb128087) were obtained from Biorbyt Ltd. (Cambridge, UK). A specific antibody against 5-LO (#10007820) and EIA kits for LTC_4_, LTB_4_ and PGE_2_ were obtained from Cayman Chemical Co. (Ann Arbor, MI, USA). ELISA kits for IL-4 and TNF-α were purchased from R&D systems, Inc. (Minneapolis, MN, USA). Minimum Eagle’s medium (MEM) and fetal bovine serum (FBS) were from the American Type Culture Collection (Manassas, VA, USA). All other chemicals and reagents used were of analytical grade.

### 3.2. Plant M aterial and Preparation of ETZL and Tricin

The aerial parts of *Z. latifolia* were purchased from the Pureunsan Agricultural Association Corporation (Dongdaemun-gu, Seoul, Korea) and ETZL was provided by the BTC Corporation (Sangnok-gu, Ansan, Korea). Briefly, the dried leaves of *Z. latifolia* were incubated with mixed hydrolysis enzymes (cellulase, hemicellulase and pectinase) at 35 °C for 16 h in H_2_O, followed by heating to inactivate the enzymes. The extracted solution was filtered and acquired. After extraction of the residue with 70% ethanol (Duksan Science, Seoul, Korea) at 80 °C for 6 h, the filtered and extracted solution was mixed with the first enzyme extract and the total enzyme extract was concentrated and dried to produce ETZL. Tricin was isolated from ETZL as previously described [8,20]. The isolated tricin was reanalyzed by HPLC and was > 99% pure. 

### 3.3. Cell Culture

RBL-2H3 cells, a mast cell line originating from rat basophilic leukemia, were cultured in MEM medium supplemented with 10% (*v*/*v*) FBS, 100 U/mL penicillin and 100 μg/mL streptomycin, at 37 °C in a humidified atmosphere of 5% CO_2_. All experiments contain a control group as the vehicle control group, comprising 0.1% DMSO.

### 3.4. Cytotoxicities of Tricin and ETZL in RBL-2H3 Cells

Briefly, the RBL-2H3 cells were seeded in a 24-well plate (1 × 10^5^ cells/well) in MEM medium supplemented with 10% FBS, and cultured overnight at 37 °C. The cells were washed with 1 × PBS, and subsequently incubated with anti-DNP-IgE (0.05 μg/mL) for 24 h. The IgE-sensitized cells were incubated with varying concentrations of tricin or ETZL (0–500 ng/mL or 0–500 μg/mL, respectively) for 1 h. DNP-HSA (0.1 μg/mL) was then added to the wells, and the mixture was incubated for a further 4 h. To measure cell viability, the absorbance was measured at 550 nm using a well-plate multi-reader.

### 3.5. Regulation of β-Hexosaminidase Releases of ETZL and Tricin in IgE-Stimulated RBL-2H3 Cells

The RBL-2H3 cells were incubated in a 24-well plate (1 × 10^5^ cells/well), overnight at 37 °C. Cultured cells were washed with 1 × PBS, and subsequently incubated with anti-DNP-IgE for 24 h. IgE-sensitized cells were exposed to varying concentrations of tricin or ETZL (0–500 ng/mL or 0–500 μg/mL, respectively) for 1 h, spiked with DNP-HSA and then incubated for a further 4 h. To measure the amount of β-hexosaminidase activity released from the cells, the culture medium was transferred and centrifuged (13,000× *g* for 10 min) at 4 °C. The supernatant (25 μL) was mixed with 50 μL p-NAG (10 mM) in 0.1 M sodium citrate buffer (pH 4.5) in a 96-well plate, and then incubated for 1 h at 37 °C. The reaction was terminated by adding sodium carbonate buffer (0.1 M, pH 10.0). The β-hexosaminidase activity was determined by measuring the difference in absorbance at 405 nm.

### 3.6. Effect of ETZL and Tricin on the Cytokine and Chemokine Secretions in IgE-Mediated RBL-2H3 Cells 

To measure the cytokine levels in the culture medium, all culture media were centrifuged (13,000× *g* for 10 min) at 4 °C, and the samples were stored at −80 °C until use. The concentrations of IL-4 and TNF-α were determined using ELISA kits (R&D systems, Inc.), according to the manufacturer’s instructions. To determine the levels of LTB_4_, LTC_4_ and PGE_2_ all culture media were centrifuged (13,000× *g* for 10 min) at 4 °C, and the supernatant was stored at −80 °C until use. The concentrations of LTB_4_, LTC_4_ and PGE_2_ were determined using EIA kits (Cayman Chemical, Inc., Ann Arbor, MI, USA), according to the manufacturer’s instructions.

### 3.7. Western Blot

RBL-2H3 mast cells were lysed in lysis buffer (Pro-Prep^tm^, iNtRON, Seongnam, Korea) containing protease inhibitor (P3100_001, GenDEPOT, Barker, TX, USA) and phosphatase inhibitor (P3200_001, GenDEPOT, Baker, TX, USA), with subsequent incubation on ice for 30 min. Protein concentrations in cell lysates were determined using a BCA protein assay (Thermo Fisher Scientific, Waltham, MA, USA). Proteins (50 μg) were loaded and separated by 10% sodium dodecyl sulfate-polyacrylamide gel electrophoresis, transferred to PVDF membranes (Whatman GmbH, Dassel, Germany), blocked with 5% skim milk in Tris-buffered saline containing 0.1% Tween-20 for 1 h, and then incubated with primary antibodies for 16 h at 4 °C. After three washes in Tris-buffered saline containing 0.1% Tween-20, membranes were incubated with horseradish peroxidase-linked secondary antibodies (Cell Signaling Technology, Beverly, MA, USA) for 1 h. Proteins were detected by enhanced chemiluminescence, and visualized using image software (UVP Vision Works^®^ LS Image Acquisition & Analysis Software, Upland, CA, USA). 

### 3.8. Statistical Analysis

Data are presented as means ± SD values of three experiments. Differences among groups were examined using Student‘s *t* test, and a significant difference is considered at *p* < 0.05, *p* < 0.01 and *p* < 0.001.

## References

[1] Zhang H., Cao P., Agellon L.B., Zhai C.K. (2009). Wild rice (*Zizania latifolia* (Griseb) Turcz) improves the serum lipid profile and antioxidant status of rats fed with a high fat/cholesterol diet. Br. J. Nutr..

[2] Liu M., Qian B., Zhang H., Deng Y., Shen Y., Ping J., Cao L. (2010). Sanitizer treatments alleviate lignification of sliced few-flower wildrice (*Zizania latifolia* Turcz.). Food Res. Int..

[3] Deng Y., Luo Y., Qian B., Liu Z., Zheng Y., Song X., Lai S., Zhao Y. (2014). Antihypertensive effect of few-flower wild rice (*Zizania latifolia* Turcz.) in spontaneously hypertensive rats. Food Sci. Biotechnol..

[4] Park W.H., Cha Y.Y. (2005). Inhibition effect of *Zizania latifolia* on apoptosis induced by H_2_O_2_ in Neuro2A cell. J. Physiol. Pathol. Korean Med..

[5] Qian B., Luo Y., Deng Y., Cao L., Yang H., Shen Y., Ping J. (2012). Chemical composition, angiotensin-converting enzyme-inhibitory activity and antioxidant activities of few-flower wild rice (*Zizania latifolia* Turcz.). J. Sci. Food Agric..

[6] Moon J.M., Park S.H., Jhee K.H., Yang S.A. (2018). Protection against UVB-induced wrinkle formation in SKH-1 hairless mice: Efficacy of tricin isolated from enzyme-treated *Zizania latifolia* extract. Molecules.

[7] Park S.H., Lee S.S., Bang M.H., Jo S.K., Jhee K.H., Yang S.A. (2018). Protection against UVB-induced damages in human dermal fibroblasts: Efficacy of tricin isolated from enzyme-treated *Zizania latifolia* extract. Biosci. Biotechnol. Biochem..

[8] Lee S.S., Baek Y.S., Eun C.S., Yu M.H., Baek N.I., Chung D.K., Bang M.H., Yang S.A. (2015). Tricin derivatives as anti-inflammatory and anti-allergic constituents from the aerial part of *Zizania latifolia*. Biosci. Biotechnol. Biochem..

[9] Cai H., Al-Fayez M., Tunstall R.G., Platton S., Greaves P., Steward W.P., Gescher A.J. (2005). The rice bran constituent tricin potently inhibits cyclooxygenase enzymes and interferes with intestinal carcinogenesis in ApcMin mice. Mol. Cancer Ther..

[10] Gilfillan A.M., Tkaczyk C. (2006). Integrated signalling pathways for mast-cell activation. Nat. Rev. Immunol..

[11] Church M.K., Levi-Schaffer F. (1997). The human mast cell. J. Allergy Clin. Immunol..

[12] Wills-Karp M. (1999). Immunologic basis of antigen-induced airway hyperresponsiveness. Annu. Rev. Immunol..

[13] Kitaura J., Asai K., Maeda-Yamamoto M., Kawakami Y., Kikkawa U., Kawakami T. (2000). Akt-dependent cytokine production in mast cells. J. Exp. Med..

[14] Pastorello E.A., Ortolani C., Farioli L., Pravettoni V., Ispano M., Borga Å., Bengtsson A., Incorvaia C., Berti C., Zanussi C. (1994). Allergenic cross-reactivity among peach, apricot, plum, and cherry in patients with oral allergy syndrome: An in vivo and in vitro study. J. Allergy Clin. Immunol..

[15] Frossi B., Rivera J., Hirsch E., Pucillo C. (2007). Selective activation of Fyn/PI3K and p38 MAPK regulates IL-4 production in BMMC under nontoxic stress condition. J. Immunol..

[16] Yoo J.M., Park E.S., Kim M.R., Sok D.E. (2013). Inhibitory effect of N-Acyl dopamines on IgE-mediated allergic response in RBL-2H3 cells. Lipids.

[17] Yoo J.M., Yang J.H., Kim Y.S., Cho W.K., Ma J.Y. (2016). Inhibitory effect of *Loranthus parasiticus* on IgE-mediated allergic responses in RBL-2H3 Cells. Mediators Inflamm..

[18] Lee S.S., Baek N.I., Baek Y.S., Chung D.K., Song M.C., Bang M.H. (2015). New flavonolignan glycosides from the aerial parts of *Zizania latifolia*. Molecules.

[19] Jin S.E., Ha H.K., Shin H.K., Seo C.S. (2019). Anti-allergic and anti-inflammatory effects of Kuwanon G and Morusin on MC/9 mast cells and HaCaT keratinocytes. Molecules.

[20] Kim D.E., Min K.J., Kim M.J., Kim S.H., Kwon T.K. (2019). Hispidulin inhibits mast cell-mediated allergic inflammation through down-regulation of histamine release and inflammatory cytokines. Molecules.

[21] Lee M., Shim S.Y. (2020). Inhibitory effects of eriodictyol-7-O-β-d-glucuronide and 5, 7-dihydroxy-4-chromene isolated from *Chrysanthemum zawadskii* var. latilobum in FcεRI-mediated human basophilic KU812F cell activation. Molecules.

[22] Quan G.H., Chae H.S., Song H.H., Ahn K.S., Lee H.K., Kim Y.H., Oh S.R., Chin Y.W. (2013). Anti-allergic flavones from *Arthraxon hispidus*. Chem. Pharm. Bull..

[23] Park E.J., Park K.C., Eo H., Seo J., Son M., Kim K.H., Chang T.S., Cho S.H., Min K.U., Jin M. (2007). Suppression of spontaneous dermatitis in NC/Nga murine model by PG102 isolated from *Actinidia arguta*. J. Invest. Dermatol..

[24] Kim D., Kim S.H., Park E.J., Kim J., Cho S.H., Kagawa J., Arai N., Jun k., Kiyono H., Kim S. (2009). Suppression of allergic diarrhea in murine ovalbumin-induced allergic diarrhea model by PG102, a water-soluble extract prepared from *Actinidia arguta*. Int. Arch. Allergy Immunol..

[25] Kim D., Kim S.H., Park E.J., Kang C.Y., Cho S.H., Kim S. (2009). Anti-allergic effects of PG102, a water-soluble extract prepared from *Actinidia arguta*, in a murine ovalbumin-induced asthma model. Clin. Exp. Allergy.

[26] Mastuda H., Morikawa T., Ueda K., Managi H., Yoshikawa M. (2002). Structural requirements of flavonoids for inhibition of antigen-induced degranulation, TNF-α and IL-4 production from RBL-2H3 cells. Bioorg. Med. Chem..

[27] Itoh T., Ninomiya M., Nozawa Y., Koketsu M. (2010). Chalcone glycosides isolated from aerial parts of *Brassica rapa* L.‘hidabeni’suppress antigen-stimulated degranulation in rat basophilic leukemia RBL-2H3 cells. Bioorg. Med. Chem..

[28] Razali N.A., Nazarudin N.A., Lai K.S., Abas F., Ahmad S. (2018). Curcumin derivative, 2, 6-bis (2-fluorobenzylidene) cyclohexanone (MS65) inhibits interleukin-6 production through suppression of NF-κB and MAPK pathways in histamine-induced human keratinocytes cell (HaCaT). BMC Complement. Altern. Med..

[29] Nanda B.L., Nataraju A., Rajesh R., Rangappa K.S., Shekar M.A., Vishwanath B.S. (2007). PLA_2_ mediated arachidonate free radicals: PLA_2_ inhibition and neutralization of free radicals by anti-oxidants-a new role as anti-inflammatory molecule. Curr. Top. Med. Chem..

[30] Shalini V., Bhaskar S., Kumar K.S., Mohanlal S., Jayalekshmy A., Helen A. (2012). Molecular mechanisms of anti-inflammatory action of the flavonoid, tricin from Njavara rice (*Oryza sativa* L.) in human peripheral blood mononuclear cells: Possible role in the inflammatory signaling. Int. Immunopharmacol..

[31] Koyasu S. (2003). The role of PI3K in immune cells. Nat. Immunol..

[32] Kim H.K., Kim J.W., Zilberstein A., Margolis B., Kim J.G., Schlessinger J., Rhee S.G. (1991). PDGF stimulation of inositol phospholipid hydrolysis requires PLC-γ1 phosphorylation on tyrosine residues 783 and 1254. Cell.

[33] Cho S.H., Woo C.H., Yoon S.B., Kim J.H. (2004). Protein kinase Cδ functions downstream of Ca^2+^ mobilization in FcεRI signaling to degranulation in mast cells. J. Allergy Clin. Immunol..

[34] Han E.H., Park J.H., Kim J.Y., Chung Y.C., Jeong H.G. (2009). Inhibitory mechanism of saponins derived from roots of *Platycodon grandiflorum* on anaphylactic reaction and IgE-mediated allergic response in mast cells. Food Chem. Toxicol..

